# Supplementary material for: The role of brain oscillations in predicting self-generated sounds
Source: Neuroimage. 2017 Feb 15;147:895–903. doi: 10.1016/j.neuroimage.2016.11.001 (PMC5315057; doi:10.1016/j.neuroimage.2016.11.001)
Supplement: Supplementary file 1 — Supplementary material [file mmc1.docx]

**Supporting Information for**

‘The role of brain oscillations in predicting the sensory consequences of your actions’ by

Liyu Cao, Gregor Thut, and Joachim Gross

**Robust correlation results using Spearman skipped correlations (40).**

Between SA and pre-stimulus alpha power increase: Spearman’s rho = -0.65, 95% CI = [-0.93 -0.12];

Between SA and post-stimulus alpha phase locking decrease: Spearman’s rho = 0.89, 95% CI = [0.78 0.98];

Between SA and post-stimulus high gamma power decrease: Spearman’s rho = 0.67, 95% CI = [0.17 0.92];

Between SA and post-stimulus low gamma power decrease: Spearman’s rho = 0.65, 95% CI = [0.18 0.93];

Between pre-stimulus alpha power increase and post-stimulus high gamma power decrease: Spearman’s rho = -0.80, 95% CI = [-0.95 -0.41];

Between post-stimulus alpha phase locking decrease and post-stimulus high gamma power decrease: Spearman’s rho = 0.73, 95% CI = [0.31 0.91].

**Supplementary Figures**

**
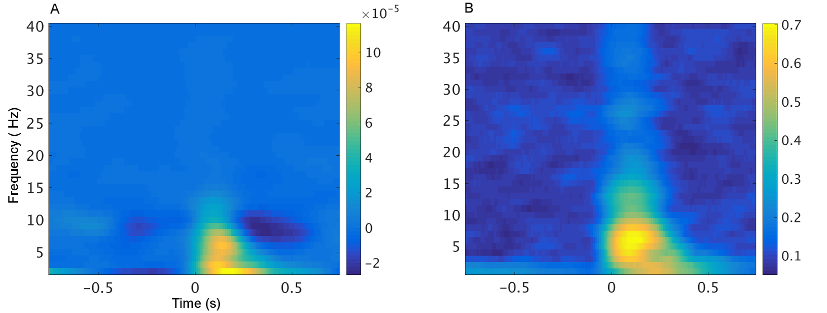
**

**Figure S1** The average power and phase locking induced by the stimulus shown with data from the analysed right auditory voxel in the passive jittered condition. The power is baseline corrected to the pre-stimulus time window (-750 to 0 ms).


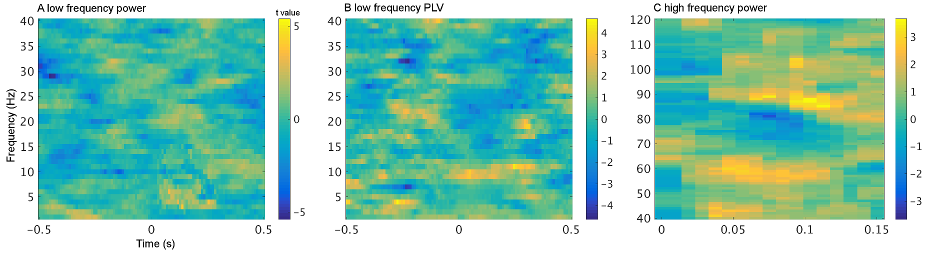


**Figure S2** Correlation maps between SA and low frequency power changes (A), between SA and low frequency phase locking value changes (B), between SA and high frequency power changes (C). The correlation maps are obtained with Spearman correlation as implemented in ‘ft_statfun_correlationT’.


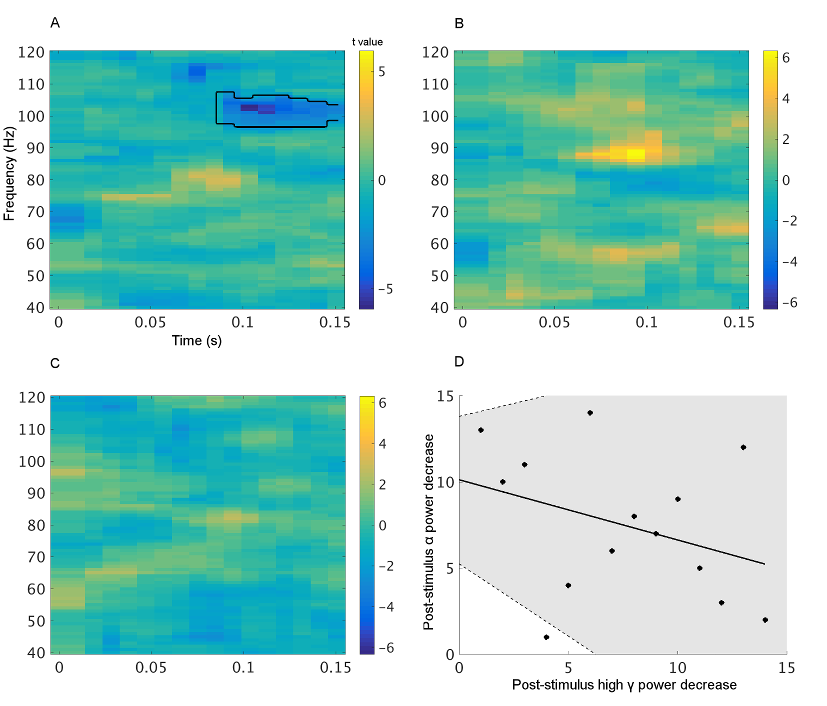


**Figure S3** Correlation maps between the oscillatory changes across participants. (A) shows the correlation between pre-stimulus alpha power increase and post-stimulus gamma power changes across time and frequency; (B) shows the correlation between post-stimulus alpha phase locking decrease and post-stimulus gamma power changes across time and frequency. (C) shows the correlation between post-stimulus alpha power decrease and post-stimulus gamma power changes over time and frequency; (D) shows the scatter plot between post-stimulus alpha power decrease and post-stimulus high gamma power decrease (Spearman’s rho = -0.35, p = 0.22, CI = [-0.95 0.30]). The correlation maps were obtained with Spearman correlation as implemented in ‘ft_statfun_correlationT’.


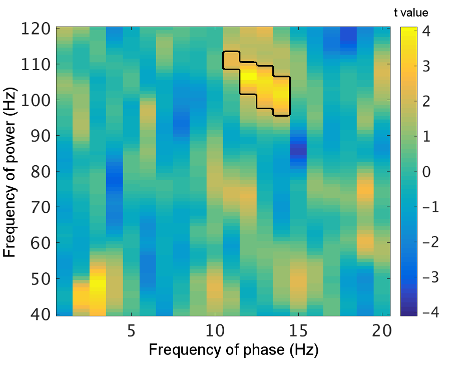


**Figure S4** The post-stimulus gamma power is only significantly correlated with post-stimulus alpha/beta phase. The correlation was first computed between post-stimulus phase deviation (70-160 ms) and post-stimulus gamma power (0-40) ms for each time-frequency combination. Then the average over the time doamin was compared to the same correlation but calculated with a baseline gamma power (-300 to 0 ms). Fisher z transform was applied before paired t-tests with cluster correction.
